# Well-being and mathematics achievement: What is the role of gender, instructional clarity, and parental involvement?

**DOI:** 10.3389/fpsyg.2022.1044261

**Published:** 2023-01-19

**Authors:** Angelina Wilson Fadiji, Vijay Reddy

**Affiliations:** ^1^Department of Educational Psychology, Faculty of Education, University of Pretoria, Pretoria, South Africa; ^2^Africa Unit for Transdisciplinary Health Research, Faculty of Health Sciences, North-West University, Potchefstroom, South Africa; ^3^Inclusive Economic Development, Human Sciences Research Council, Durban, South Africa

**Keywords:** well-being, mathematics achievement, instructional clarity, parental involvement, South Africa, education quality and outcomes

## Abstract

**Introduction:**

The aim of the present study was to explore the relationship between satisfaction with life and mathematics achievement among a nationally representative sample of Grade 9 learners in South Africa.

**Methods:**

Using the Trends in Mathematics and Science Study (TIMSS 2019) based on a sample of 20,829 learners (females = 11,067 and males = 9,719), and employing structural equation modelling (SEM), we examined the nature of the relationship between satisfaction with life and mathematics achievement, considering the role of gender, parental involvement, and instructional clarity.

**Results:**

Our findings showed that satisfaction with life is positively related to mathematics achievement, but is not moderated by gender. Additionally, instructional clarity contributes to, and is a partial mediator of, the relationship between life satisfaction and mathematics achievement. This suggests that greater instructional clarity is positively associated with high achievement in mathematics, over and above the relationship with satisfaction with life. By contrast, parental involvement negatively mediates this relationship, suggesting that mathematics achievement is negatively associated with certain forms of parental involvement, such as setting time aside for homework; and checking if homework is done.

**Discussion:**

Given the tested linear relationship between life satisfaction, instructional clarity and mathematics achievement, the results of the study suggest that if wellbeing is improved and existing instructional practices are constantly reviewed, mathematics scores could be positively affected. In addition, the emerging finding on the negative role of parental involvement in the hypothesised mediated relationship suggests that learners could benefit from properly tailored, and government-sponsored, afterschool tutoring.

## Introduction

In South Africa, the focus of education research has been on academic achievement and how home, school and classroom factors affect achievement—without detailed consideration of how individual wellbeing may play a role. The current evidence on predictors of academic achievement in South Africa also focuses primarily on self-efficacy (Juan et al., 2018), learning experiences (Visser et al., 2019), socio-economic status (SES) and school resources (Juan and Visser, 2017), among others. However, for the first time, as part of the Trends in Mathematics and Science Study (TIMSS) national dataset, data on subjective wellbeing indicators (satisfaction with life, one indicator of wellbeing) was gathered from Grade 9 learners to investigate the relationship between a wellbeing indicator and mathematics achievement. To further understand the nature of this relationship, the current study asks for whom this relationship is significant (males or females) and under what external circumstances (i.e., instructional clarity and parental involvement). The latter are key factors in the learning process and they cover both what happens at school and home.

Previous studies have painted an inconsistent picture of the relationship between gender and subjective wellbeing- defined as the satisfactory evaluation of life, presence of positive emotion and absence of negative affect (Wilson and Somhlaba, 2018). While some studies found no gender differences (Batz and Tay, 2018), others have pointed to females experiencing greater subjective wellbeing (Graham and Chattopadhyay, 2013; Tay et al., 2014). With respect to the lack of gender differences, Batz and Tay (2018) opined that men and women adapt to their surroundings and habitual conditions of living (for instance inequality), making differences in wellbeing either small or non-existent (see also Hyde, 2005). Females experiencing more subjective wellbeing may be linked to social norms that allow women to be more emotionally expressive whether positive or negative emotions (Simon and Nath, 2004).

Furthermore, in South Africa, and some parts of the world, females tend to experience less favourable objective conditions of wellbeing (such as, material resources), which could have implications for their subjective wellbeing and associated life domains (Joshanloo and Jovanović, 2020), including academic achievement. Given the inconsistent findings on gender and wellbeing, it will be interesting to understand the extent to which gender might moderate the relationship between subjective wellbeing and mathematics achievement. Previous research has also shown that school characteristics and parental involvement are important for learners’ educational achievement (Bryce et al., 2019). Such a relationship is built on the premise that parents and teachers could provide different forms of support to enable learning. Therefore, in this study, we also seek to address the question of which (gender), and under what circumstances wellbeing might be beneficial for mathematics achievement in our attempt to explore the potential benefits (if any) of promoting wellbeing in South African schools.

### Subjective wellbeing and academic achievement

Both subjective wellbeing and academic achievement are indicators of positive psychological functioning (Suldo et al., 2006), and, according to the OECD (2017), these are variables found in a high-performing education system. Successful students not only perform well academically, they are also satisfied at school, probably owing to a mutually reinforcing relationship (OECD, 2017). That is, when students are well, they tend to succeed academically and vice versa (Zakaria and Abdul Halim, 2017). Support for this statement is underscored by schools being not only a place for academic learning, but also for social interaction, personality development and becoming acquainted with larger society (Bücker et al., 2018). Subjective wellbeing and academic achievement constitute the essence of educational endeavours and are mutually dependent.

Following the finding on subjective wellbeing predicting academic performance among a sample of Mexican students (Ojeda et al., 2011; Whitley et al., 2012), the authors argued that positive wellbeing experiences expand psychological resources, such as the intellectual ability needed for academic success. More so, greater subjective wellbeing might be linked to higher levels of vigour in school activities, and positive emotions while completing academic tasks, which in turn might lead to excelling in academic endeavours (see Cohn and Fredrickson, 2009). Another explanation for the relationship between satisfaction with life and academic achievement is that experiencing such optimal psychological state enables learners to broaden their thought-action repertoire, which is deemed essential for acquiring durable academic resources (Fredrickson, 2013).

Among a group of undergraduate Filipino students, wellbeing predicted objective academic achievement (after controlling for relevant demographic variables) (Datu, 2018). This is in line with the positive education paradigm (a dual emphasis on wellbeing and achievement in schools), as it posits that wellbeing is beneficial because it cultivates different indicators of academic success. Similarly, in a group of USA students, Heffner and Antaramian (2016) found that greater life satisfaction predicted increased academic achievement among 7th and 8th Graders at an average age of 13 years. In contrast to Heffner and Antaramian (2016), a meta-analysis of 47 studies including 151 effect sizes, Bücker et al. (2018) showed that low-achieving learners do not necessarily have lowered subjective wellbeing, and learners scoring low on wellbeing were not necessarily low achieving. However, they did find small to moderate significant correlations between academic achievement and subjective wellbeing. Despite the relatively weak degree of association between wellbeing and academic outcomes, Noftle and Robins (2007) note that even empirical investigations with small effect sizes may have valuable practical impact, because of the importance attached to experiencing wellbeing. The claims of inconsistent findings (see Bücker et al., 2018) in the relationship between subjective wellbeing and academic achievement warrant further exploration (Heffner and Antaramian, 2016).

### Gender, subjective wellbeing, and academic achievement

Evidence on gender differences in subjective wellbeing (Bradshaw et al., 2011) is also unclear for a number of reasons. For example, whereas some studies have supported the notion that females have a higher tendency to report happiness (Tomyn and Cummins, 2011; Casas et al., 2013; Cummins, 2014), the converse has been found in studies such as by Lyons et al. (2014) that males have greater wellbeing. Still others (Proctor et al., 2009) suggest that there are no gender differences. One of the reasons for this inconsistency is the use of multidimensional versus general overall satisfaction with life measures (Dinisman and Ben-Arieh, 2016), where single-item measures shows no difference but multidimensional assessment tools demonstrates some differences because males and female might have unique experiences when it comes certain domains of wellbeing. For instance, females might do better on satisfaction with interpersonal relationships (Casas et al., 2013).

Another factor that may explain the lack of a consistent pattern between subjective wellbeing and gender is the different gender roles (Eagly and Karau, 2002). That is, expectations and norms in gender roles influence the perceptions regarding the appropriate occupations for men and women, the type of traits men and women should possess, and even the type of emotions that are acceptable to experience and demonstrate for men and women (Eagly and Karau, 2002), all of which can impact on wellbeing. Meisenberg and Woodley (2015) contend that socio-cultural conditions moderate the correlation between gender and life satisfaction. Specifically, the African tradition and culture have socialised boys and girls into gender roles which might have implications for wellbeing.

In a similar vein, males and females may have different educational experiences. Regarding academic achievement, a meta-analysis by Voyer and Voyer (2014) found that female students generally earn higher grades than male students. In South Africa, a similar finding of female nursing students academically outperforming males emerged in a systematic review (Mthimunye and Daniels, 2019). One explanation offered for this trend is the tendency for parents to encourage females to exert greater effort in their studies, while assuming that males do not require extra assistance to succeed in their academics (Voyer and Voyer, 2014). Research by Varner and Mandara (2014), for example, confirmed this suggestion among a sample of African American adolescents, concluding that “reducing gaps in parenting may help reduce the gender gap in achievement (p. 12).”

Pertaining to the possible moderating role of gender, in the meta-analyses by Bücker et al. (2018), gender played an insignificant role in the relationship between subjective wellbeing and academic achievement. Similarly, Cadime et al. (2016) found no gender difference in the relationship between academic achievement and wellbeing. It was suggested that because children and young adults spend a substantial amount of their time at school, students’ overall life satisfaction and their academic satisfaction might be highly overlapping and not as distinct as expected (Bücker et al., 2018).

On the one hand, other evidence has however shown that existing gender difference in this relationship might be attributed to internal distress that girls may experience, while evaluating themselves more negatively than boys, and being more prone than boys to worry about their performance at school (Pomerantz et al., 2002). On the other hand, girls doing better than boys may be as a result of them compensating for lower wellbeing with a higher readiness to perform well in school. This phenomenon has been referred to as a higher “conformity of girls towards school requirements” (e.g., Sparfeldt et al., 2009). However, the evidence of the moderating role of gender is still inconclusive.

### Role of instructional clarity and parental involvement

The teacher-learner relationship is an important factor of the classroom climate. Instructional clarity refers to the teacher’s ability to explain content clearly to students and to provide clear directions during teaching. Moreover, trusting, respectful and caring interactions between the teacher and learner provide the emotional and intellectual context for engagement and related educational outcomes (Ryan and Deci, 2002; Guthrie et al., 2006). Previous reviews have shown that teachers’ instructional practices may have an impact on students’ learning and may be more important than class size and classroom climate—even more important than the teacher’s years of experience and formal training (Seidel and Shavelson, 2007; Timperley and Alton-Lee, 2008; Konstantopoulos and Chung, 2011). It is hence important for the teacher to adopt instructional tools and techniques that guarantee students’ clear understanding of tasks (Arends, 2021).

Instructional practices in mathematics were found to predict achievement among upper and middle elementary learners in the USA (Kane and Staiger, 2012; Blazar and Kraft, 2017). Yagan (2021), for example, found a significant relationship between teachers’ classroom management and instructional clarity skills on the one hand, and students’ mathematics achievement and attitude toward mathematics on the other. These studies support the notion that quality teaching practices have the potential to positively influence achievement (Fischer and Neumann, 2012), especially in contexts where minimal conditions for learning are in place. Additionally, teacher clarity has been found to influence positive outcomes with regard to students’ academic motivation and critical thinking (Loes and Pascarella, 2015), as well as their affective learning (Chesebro and McCroskey, 2001; Titsworth et al., 2015).

The relationship between instructional clarity and mathematics achievement is underscored by a number of factors. First, positive teaching and learning practices are argued to increase academic achievement because they facilitate learning and engagement (Osher and Kendziora, 2010; Thapa et al., 2013; Lee et al., 2017). Second, the degree of explicitness, clarity of learning goals, and content-oriented instructions (when in place) provide a clear pathway for learning to occur (Klette et al., 2017). Third, high teacher expectations provide motivation for learners to exert the necessary effort at ensuring improved achievement (Graham et al., 2017). Despite the evidence provided, not much of this has been replicated in the South African context; hence this study.

Education researchers have always expressed interest in the relationship between parental involvement and academic achievement (Fan and Chen, 2001). Parents involved in a learner’s education contribute to their child’s social, emotional, and intellectual growth (Green et al., 2007). Despite this, some inconsistency still surrounds the existing findings on parental involvement and its association with students’ academic achievement (McNeal, 2012). This is mostly attributed to a lack of conceptual clarification and measurement of parental involvement (Bakker and Denessen, 2007; Wilder, 2014). For instance, parental involvement is usually associated with investment in education, provision of resources (LaRocque et al., 2011) and actual learning support for children by their caregivers. It could also include providing support for education at home and engagement with teachers at school (Comer, 1995).

A systematic review of 75 studies conducted between 2003 and 2017 revealed that several parental involvement indicators, including parental modelling, expectations, encouragement and school involvement, are related to academic achievement (Gubbins and Otero, 2016; Boonk et al., 2018). Parental encouragement, for example, has been shown to foster student academic achievement through letting children know parents care about them and their performance (Rogers et al., 2009). Adding to this, is the provision of an appropriate learning environment at home to support homework and learning (Sheldon and Epstein, 2005; Gonida and Cortina, 2014).

In contrast, Boonk et al. (2018) argue that not all parental involvement fosters academic achievement. Specifically, involvement in homework was found to have either a negative relationship (Lee and Bowen, 2006) or no relationship (Driessen et al., 2005) in American and Dutch samples when parents lacked the requisite skills. Other evidence also indicates that the nature of homework involvement (autonomy support, control, interference, cognitive engagement) determines its relationship with achievement (Gonida and Cortina, 2014). For instance, when parents are trained to assist learners (Tam and Chan, 2009) and there is some level of autonomy (Gonida and Cortina, 2014), then parental involvement positively predicts achievement. In South Africa, according to TIMSS reports (Reddy et al., 2022), (while being cautious of high missing values) only 38% of households have at least one parent with a post-secondary education, implying that the benefits of involvement in learners’ education might not be so apparent for the greater sample of learners as a result of the educational level of parents. As a result, this study tests the nature of relationship between parental involvement and mathematics achievement.

### The present study

As psychologists increase their use of strengths-based assessments, knowing the role of subjective wellbeing beyond that of other affective states (such as happiness) alongside school functioning may help inform educational practice. In this study, we explore whether this association is moderated by gender and further mediated by parental involvement and instructional clarity. The moderating and mediating variables were introduced because of evidence on their relationship with achievement. We used structural equation modelling (SEM) in this paper because it allows for the measurement of a structural relationship and estimates the multiple and interrelated dependence of variables in a single analysis. This relationship includes the direct relationships between life satisfaction, parental involvement and instructional clarity and mathematics achievement, the moderation by gender and mediation by parental involvement and instructional clarity. Since the technique allows for the measurement of several predictors of mathematics achievement in one model, our study will be a step in the direction of attempting to promote academic achievement alongside subjective assessments of the quality of life of South African learners.

### Research hypothesis

1. There will be a direct positive relationship between satisfaction with life and mathematics achievement.
2. Gender will moderate the relationship between satisfaction with life and mathematics achievement.
3. Parental involvement and instructional clarity is a positive predictor of mathematics achievement.
4. Parental involvement and instructional clarity mediate the relationship between satisfaction with life and achievement in mathematics.

## Methodological approach

The TIMSS 2019 assessment was its seventh cycle in South Africa. The cycles have been conducted every 4 years since 1995. To inform educational policy in the participating countries, TIMSS also collects extensive background information on the home and school contexts in which teaching and learning take place. This background information is collected through a series of questionnaires for learners, parents, mathematics and science educators, school principals and curriculum specialists.

South African learners who participated in TIMSS 2019 completed a paper-based assessment booklet containing an even distribution of both mathematics and science items. These booklets were designed to be administered in two sessions, separated by a short break. Each session was 45 min in duration. In addition to completing the achievement booklet, each learner also completed a background questionnaire.

The Learner Questionnaire asks about aspects of learners’ home and school lives, their home environment, their school climate for learning, and their perceptions of and attitudes toward mathematics and science. Local items about satisfaction of life were included in the Learner Questionnaire specific to South Africa alone.

### Sample and participants

To conduct TIMSS in South Africa, a representative sample was drawn from schools offering Grade 9 classes. TIMSS 2019 followed the sampling procedures described in the International Association for the Evaluation of Educational Achievement (IEA) TIMSS Methods and Procedures Manual (Martin et al., 2020). In the two-stage stratified cluster sampling design, schools were randomly selected at the first stage and an intact Grade 9 class was selected at the second stage.

A total of 524 schools were selected for the study, of which 520 participated. This sample is higher than in most countries, as South Africa over-sampled schools in two provinces, Gauteng and Western Cape, to be able to better estimate the provincial achievement scores (Reddy et al., 2022).

This sample included 20,829 learners (females = 11,067 and males = 9,719), with a higher number of learners from the Gauteng and Western Cape Provinces. Weights allocated to each learner ensured that the national sample was representative of the Grade 9 South African population.

### Data gathering and ethical considerations

The main survey was administered by an external data collection agency with relevant qualifications and experience in the field of data collection. Grade 9 learners took part in the assessment in August 2019. The Human Sciences Research Council (HSRC) Ethics Committee approved the study (REC/4/16/03/11).

### Instruments

In this study, the following items are included from the Grade 9 TIMSS 2019 dataset: Satisfaction with life, mathematics achievement, instructional clarity, parental involvement, and gender.

#### Satisfaction with life

In order to measure subjective wellbeing, four items on the Satisfaction with Life Scale (SWLS) were adapted and used (Diener et al., 1985). Items used to compute this index included satisfaction with school, peers, home, and life as a learner. Items were scored on a scale ranging from 1 = *Highly dissatisfied* to 4 = *Highly satisfied*. Higher scores indicated greater satisfaction with life.

#### Mathematics achievement

The value for achievement was computed using the IEA IDB analyser, combining five plausible values. Plausible values are not intended to be estimates of individual student scores. Instead, they are imputed scores for students with similar response patterns and background characteristics in the sampled population that may be used to estimate population characteristics correctly. However, the limitation is that they are generally biased estimates of the proficiencies of the individuals with whom they are associated. Thus, taking the average of the plausible values will still not yield suitable estimates of individual student scores (Martin et al., 2016). On the other hand, using plausible values in analysis provides unbiased estimates of the student population.

#### Instructional clarity in mathematics

To measure instructional clarity, some of the items reported by learners were: understanding what the teacher expects; teacher is easy to understand; teacher explains again. Items on the scales were scored as 1 = *Agree a lot* to 4 = *Disagree a lot*. In order to ensure that greater scores reflect the dimension being measured, we had to reverse score the items.

#### Parental involvement

This scale measured the extent to which parents are involved in learner’s schoolwork. Examples of questions on this scale included talking about schoolwork; setting time aside for homework; and checking if homework is done. Items on this scale were scored from 1 = *Every or almost every day* to 3 = *Never or almost never*. Items were reverse scored to ensure that greater values reflected greater parental involvement. This was with the exception of the item “Homework is too difficult for parents,” which did not need to be recoded.

### Data analysis

The IEA IDB analyser was used in conjunction with the Statistical Package for Social Sciences (IBM, SPSS) to compute descriptive statistics and correlations. Mplus software (Muthén and Muthén, 1998-2019) was also used for analysis related to structural equation modelling. Descriptive statistics, including means and standard deviation of each variable, were computed and a summary is available in Table 1. Next, inter-correlations across the variables were performed as this is a prerequisite for structural equation modelling. Cronbach alphas were also computed to assess the reliability of the composite measures of satisfaction with life, parental involvement, and instructional clarity (see Table 1).

**TABLE 1:** Summary of correlations and means by gender.

	Maths	Sat	InsClar	ParInv
Maths	1			
Sat	0.29**	1		
InsClar	0.17**	0.13**	1	
ParInv	−0.27**	−0.08**	0.15**	1
Cronbach alpha	–	0.79	0.84	0.68
Mean (SD) girls	392.77 (76.52)	3.01 (0.81)	10.74 (1.73)	3.14 (0.55)
Mean (SD) boys	386.23(77.16)	2.86 (0.86)	10.81(1.67)	3.16 (0.53)

ParInv, parental involvement; InsClar, instructional clarity; Sat, satisfaction with life; Maths, mathematics achievement; **P > 0.01.

**FIGURE 1:**
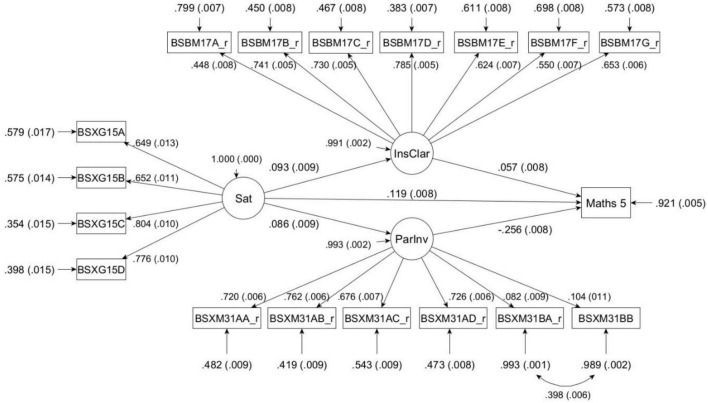
Mediated structural model. Sat, satisfaction with life; InsClar, instructional clarity; Maths 5, 5th plausible value for mathematics achievement; ParInv, parental involvement.

Two steps were taken to determine whether the observed data fit the model. First, a measurement model was used to determine the extent to which the indicators loaded strongly on their respective latent variables (satisfaction with life; instructional clarity; parental involvement; see Figure 1).

**FIGURE 2:**
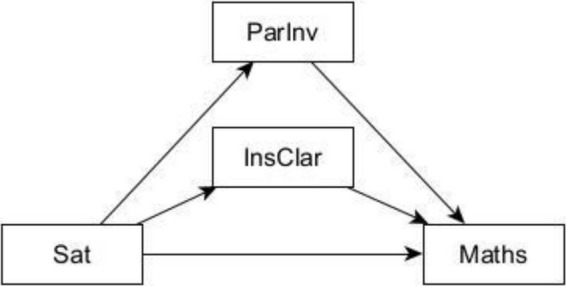
Conceptual model of the potential mediating effects of instructional clarity and parental involvement on the relationship between satisfaction with life and mathematics achievement. ParInv, parental Involvement; InsClar, instructional clarity; Sat, satisfaction with life; Maths, mathematics achievement.

This was followed by testing the direct relationships between mathematics achievement and each of the latent variables—life satisfaction, instructional practices and parental involvement—within a structural model (see Figure 2).

In addition to the chi-square value (χ^2^-value), the Comparative Fit Index (CFI), Tucker–Lewis Index (TLI), root mean square error of approximation (RMSEA), and the standardised root mean square residual (SRMR) were reported. The criteria for determining if a model has good fit indices were determined by the following cut-off points: CFI and TLI values above 0.90 indicated reasonable model fit and values above 0.95 indicated good model fit. For the RMSEA and SRMR, values smaller than 0.08 indicated reasonable model fit, and values below 0.05 good model fit (Hu and Bentler, 1999; Byrne, 2012). In addition to model fit indices, the direct and the path coefficients of the hypothesised relationships were provided. The ML estimator was used in MPLUS.

### Moderation analysis

**FIGURE 3:**
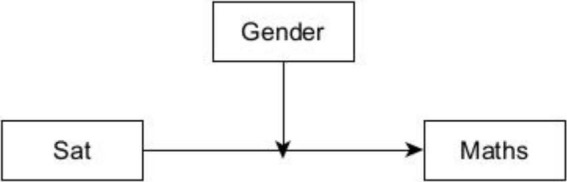
Conceptual model of the potential moderating effect of gender on the relationship between satisfaction with life and mathematics achievement. Sat, satisfaction with life; Maths, mathematics achievement.

**FIGURE 4:**
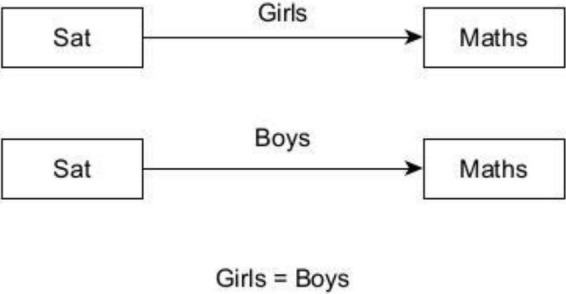
Statistical model of the potential moderating effect of gender on the relationship between satisfaction with life and mathematics achievement. Sat, satisfaction with life; Maths, mathematics achievement.

Following the testing of the direct relationships, a structural model comprising satisfaction with life and achievement, as moderated by gender, was tested using the Wald Test of Parameter of Constraints (see Figures 3, 4). A moderating factor may strengthen, diminish, negate, or otherwise alter the association between independent and dependent variables. Moderation analysis was performed using the multigroup approach with latent and observed variables. A statistically significant *p*-value of the Wald test indicates moderation as well as a relationship between satisfaction with life and mathematics achievement.

### Mediation analysis

**FIGURE 5:**
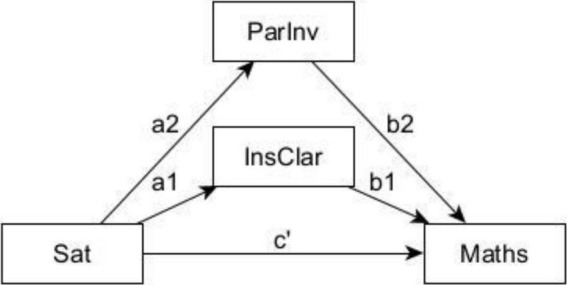
Statistical model of the potential mediating effects of instructional clarity and parental involvement on the relationship between satisfaction with life and mathematics achievement. ParInv, parental Involvement; InsClar, instructional clarity; Sat, satisfaction with life; Maths, mathematics achievement.

We also tested a mediated model comprising instructional clarity and parental involvement. A mediated model seeks to identify and explain the mechanism that underlies an observed relationship between the dependent variable (achievement) and the independent variable (satisfaction with life) through a third variable (instructional clarity and parental involvement). To perform the mediation analysis, it is a requirement that the relationships between satisfaction with life and mediator (parental involvement and instructional clarity) (a′), the relationship between the mediator and mathematics achievement (b′), and the direct relationship between the life satisfaction and mathematics achievement (c′) are statistically significant (see Figure 5). Furthermore, the indirect relationship between satisfaction with life and mathematics achievement was estimated to determine the level of mediation. Full mediation is indicated by a statistically non-significant relationship between the independent and the dependent variable before the mediator is introduced. However, should this relationship be statistically significant after the mediator was introduced and if the direct effect (c′) was reduced, there is only partial mediation. Ten thousand bias-corrected bootstrapping samples with a 95% confidence interval (CI) were applied. This was done to increase the validity of the findings.

## Results

### Correlations

Our findings show that mathematics achievement was significantly positively related to both satisfaction with life and instructional clarity (Table 1). However, a negative relationship emerged between mathematics achievement and parental involvement (over 80% of learners reported that parents supported them with homework showing very little variance in the data). Satisfaction with life was positively related to instructional clarity (*r* = 0.17) but negatively to parental involvement (*r* = -0.27). See a summary of correlations in Table 1. Satisfaction with life, instructional clarity and parental involvement all had reasonable reliability coefficients.

### Measurement model

We tested a unidimensional model for each of the three variables: satisfaction with life, parental involvement, and instructional clarity. Satisfaction with life, which was adapted from the Satisfaction with Life Scale (Diener et al., 1985), emerged with a perfect fit index showing that the hypothesised model fits the data or sample used.

The measurement models for satisfaction with life (CFI = 1.00; RMSEA = 0.00) and instructional clarity (CFI = 0.970; RMSEA = 0.053) displayed good model fit. The CFI and RMSEA values for parental involvement (CFI = 0.934; RMSEA = 0.080) were reasonable, while the TLI (0.890) and SRMR (0.098) values were unsatisfactory and indicated that the hypothesised model did not fit our sample. This implies that the hypothesised measurement model does not accurately reflect the responses from of the sample. A high modification index (MI = 272.192) indicated a high correlation between the residual terms of items (“Homework is too difficult for parents” and “Homework is in a language parent understand”). The residual terms of these items were allowed to correlate, which produced a model with good fit (CFI = 0.976; RMSEA = 0.051). The fit indices of the measurement models are presented in Table 2, showing that the hypothesised model fits the data.

**TABLE 2:** Summary of fit indices of measurement and structural models.

Model	χ^2^	Df	*P*-value	CFI	TLI	RMSEA	LL	UL	*P*-value	SRMR
1 factor original satisfaction with life	0.182	2	0.913	1.000	1.00	0.00	0.00	0.005	0.01	0.001
1 factor original parental involvement**^a^**	440.690	8	0.00	0.976	0.955	0.051	0.047	0.055	0.01	0.018
1 factor instructional clarity	841.79	14	0.00	0.970	0.955	0.053	0.050	0.057	0.030	0.025
Full direct effects model	547.21	5	0.00	0.980	0.961	0.072	0.067	0.077	0.00	0.026
Direct effects model moderated by gender	691.954	16	<001	0.976	0.969	0.064	0.060	0.068	0.00	0.036
Full indirect effects model	7665.99	130	0.00	0.931	0.919	0.053	0.052	0.054	0.00	0.065

χ^2^, Chi square; df, degrees of freedom; *p*, probability estimate; CFI, comparative fit index; TLI, Tucker–Lewis index; RMSEA, root mean square error of approximation; LL, 90% confidence interval lower limit; UP, confidence interval upper limit; SRMR, standardised root mean square residual. ^a^Residual terms of items BSXM31BB (homework being difficult for parents) and BSXM31BA_R (whether parents understand language of schoolwork) were allowed to correlate.

### Structural model

The model fit statistics for all the structural models emerged with good fit indices (see Table 2). For mathematics achievement, the simple path coefficients (a1, b1, a2, b2, and c’) (Table 3) were statistically significant (*p* < 0.05), indicating a significant relationship with life satisfaction, parental involvement and instructional clarity. The results of the moderation model pointed to a lack of gender differences in the relationship between satisfaction with life and mathematics achievement. All indirect effects from 10,000 bootstrapping samples were also statistically significant, with the bootstrapping 95% confidence interval not including zero to improve confidence in the results of the model (see Table 5). Considering that the strength of the relationship between satisfaction and mathematics achievement was reduced when instructional clarity and parental involvement were introduced into the relationship, and further considering that these relationships were still statistically significant, we conclude that the relationship between satisfaction with life and mathematics achievement was only partially mediated by instructional clarity and parental involvement, respectively. This suggests instructional clarity and parental involvement are factors contributing to the relationship between satisfaction with life and mathematics achievement. A breakdown of the path coefficients is presented in Table 3.^1^

**TABLE 3:** Results of direct effects.

Pathway—maths1	Est	SE	Est/SE	*P*-value	95% CI
Sat-InsClar (a1)	0.093	0.009	10.222	< 0.001	(0.075; 0.111)
Sat-ParInv (a2)	0.086	0.009	9.566	< 0.001	(0.069; 0.104)
InsClar-Maths1 (b1)	0.055	0.008	6.904	< 0.001	(0.040; 0.071)
Par-Inv-Maths1 (b2)	-0.259	0.009	-30.459	< 0.001	(-0.275; -0.242)
Sat-Maths1 (c’)	0.122	0.008	14.490	< 0.001	(0.105; 0.138)

Est, estimate; SE, standard error; 95% CI, 95% confidence interval; Sat, satisfaction with life; InsClar, instructional clarity; Maths, mathematics achievement; ParInv, parental involvement.

**TABLE 4:** Results of the moderation analysis for plausible value (PV) 1 mathematics achievement.

Variable	Est	SE	Est/SE	*P*	Wald test value	*df*	*p*
Maths1					0.030	1	0.8620
Sat-Maths1 (girl)	0.099	0.010	9.449	< 0.001			
Sat-Maths1 (boy)	0.110	0.011	9.985	< 0.001			
Means sat (girl)	0.000	0.000	999.000	999.000			
Means sat (boy)	-0.084	0.014	-5.867	< 0.001			

Est, estimate; SE, standard error; *p*, probability value; *df*, degrees of freedom of the Wald-test; *p*, probability value of the Wald test; Sat, satisfaction with life; Maths, mathematics achievement.

**TABLE 5:** Standardised direct and indirect effects.

				Bootstrap 10,000 times 95% CI				
Mediation models	Estimate (Est)	Standard error (SE)	Est/SE	Lower 2.5%	Lower 5%	Estimate	Upper 5%	Upper 2.5%
**Maths1—direct effect**								
Sat-Maths1	β = 0.122*	0.008	14.490	0.105	0.108	0.122	0.136	0.139
**Maths1—indirect effects**								
Total indirect	β = -0.017*	0.002	−6.963	−0.022	−0.021	−0.017	−0.013	−0.012
Sat-InsClar-Maths1	β = 0.005*	0.001	5.860	0.004	0.004	0.005	0.007	0.007
Sat-ParInv-Maths1	β = -0.022*	0.003	-8.796	−0.027	−0.027	−0.022	−0.018	−0.017

*p < 0.05.

### Satisfaction with life and mathematics achievement

Our first research hypothesis aimed to test the relationship between satisfaction with life and mathematics achievement (see Table 3). We found that there was a significant positive relationship (β = 0.103, *p* < 0.001) after computing the average coefficient, standard error and *t*-value across the five plausible values for mathematics achievement. This means that increased satisfaction with life is linked to better mathematics achievement.

### Parental involvement and mathematics achievement

In addition to the direct relationship between satisfaction with life and mathematics achievement, we tested the role of parental involvement. We assessed the extent to which parental involvement is related to achievement. Our findings revealed a significant negative relationship (β = -0.26, *p* < 0.001) between parents’ involvement in their child’s schoolwork and mathematics achievement, suggesting that parental involvement did not result in better mathematics scores. This finding might be attributable to the lack of variance in parental involvement as 85% of learners indicated that parents were involved in their homework.

### Instructional clarity and mathematics achievement

Given the role of teachers in achievement of learners, we tested the relationship between instructional clarity and mathematics achievement. Emerging from the analysis was a positive relationship (β = 0.057, *p* < 0.001) between instructional clarity and mathematics achievement, indicating that greater scores in instructional clarity was linked to better mathematics achievement scores.

### Moderation by gender

We also examined whether the relationship between satisfaction with life and mathematics achievement may be moderated by gender. Our findings showed that the *p*-value of the Wald test was statistically non-significant in each instance, implying this relationship was not statistically significant (*p* < 0.05). That is, males and females do not differ in the relationship between satisfaction with life and achievement. The results of the moderation analysis are presented in Table 4.

### Indirect effects model

Our indirect effects model tested the relationship between satisfaction with life and mathematics achievement with instructional clarity and parental achievement as mediators. The overall model showed good fit statistics (see Table 2). The hypothesised indirect model, with instructional clarity as the mediator, was also supported by significant statistics (β = 0.484, *p* < 0.001). Significant findings emerged for parental involvement, although they suggested a negative mediated relationship (β = -0.022, *p* < 0.001) (see Table 5 and Figure 5). This implies that both instructional clarity and parental involvement affect the relationship between satisfaction with life and mathematics achievement. While instructional clarity further contributes to an increase in mathematics achievement scores, parental involvement is linked to reduced scores.

## Discussion

The aim of the present study was to determine the nature of the relationships between satisfaction with life and mathematics achievement among South African Grade 9 learners. To further explore this issue, we tested the extent to which gender might moderate this relationship, given that males and females might differ in achievement as well as wellbeing levels. Keeping in mind the likely influence of external factors, such as parental involvement and instructional clarity, we tested the mediating role of certain aspects of school and home in the relationship between satisfaction with life and mathematics achievement. Our findings showed that all three models—direct effects, moderated by gender and indirect effects—had good model fit statistics. Regarding the structural relations among the variables, satisfaction with life contributed to mathematics achievement in South Africa, with such relationships being further associated with parental involvement in learning as well as instructional clarity in schools. These findings are discussed further in accordance with the stipulated research questions.

### Direct relationship between satisfaction with life and mathematics achievement

We explored the extent to which subjective wellbeing—measured as satisfaction with life—is related to mathematics achievement. We found that among Grade 9 South African learners, greater achievement in mathematics is associated with learners’ more positive satisfaction with life. Similar to the present study, a number of studies in Europe and the USA (Suldo et al., 2006; OECD, 2017; Bücker et al., 2018) argue that wellbeing and achievement are indicators of positive psychological functioning because successful students tend to also be satisfied with their lives. Supporting this argument is the notion that schools are an environment for holistic development, over and beyond academic achievement. More so, this relationship between satisfaction with life and achievement is underscored by the fact that wellbeing experiences provide more psychological resources, such as creativity and the intellectual abilities needed for greater achievement (Yang et al., 2014; Ng et al., 2015; Heffner and Antaramian, 2016). Our findings also advocate for a dual focus on wellbeing and achievement as important outcomes for schools, given that improved wellbeing is linked to increased achievement (White and Kern, 2018; Seligman and Adler, 2018).

Previous research in South Africa used proxies of wellbeing, including self-efficacy (Van der Westhuizen, 2013) and enjoyment of science (Juan et al., 2018) as predictors of achievement. The emerging relationship between satisfaction with life and academic achievement among South African learners points to the need to prioritise wellbeing in educational interventions and policies. One of the persistent challenges in South Africa has been to improve learners’ mathematics scores. To achieve this, we suggest learning support programmes that could include wellbeing interventions aimed at improving satisfaction with life across domains of school and home. In our measurement of satisfaction with life, we considered the home, peers, school and life as a learner to provide a multidimensional assessment of satisfaction with life and how this relates to mathematics achievement. These domains provide suggestions on how interventions could be tailored within the context of South Africa.

### Indirect effects model

#### Satisfaction with life and mathematics achievement mediated by parental involvement

In addition to exploring the direct relationship between satisfaction with life and mathematics achievement, we further tested the role of parental involvement in this relationship. The indirect path showed a significant relationship, albeit negative, between mathematics achievement and satisfaction with life. This implies that parental involvement negatively impacts or reduces the positive effect of satisfaction with life on mathematics achievement. A systematic review by Boonk et al. (2018) pointed to inconsistent findings on the role of parental involvement in promoting mathematics achievement, due to the varying conceptualisations of this construct. In line with the present study, while reading at home and parental engagement in learning activities were found to improve achievement (Manolitsis et al., 2013; Crosby et al., 2015), involvement in homework did not have a significant relationship with achievement (e.g., Driessen et al., 2005) or was negatively related to achievement (Domina, 2005; Lee and Bowen, 2006; Rogers et al., 2009).

Providing support for studies indicating negative or no relationship between parental involvement and mathematics achievement, our work among Grade 9 South African learners suggests that parental involvement directed at asking about homework holds less benefit for achievement, even with the existing positive relationship between satisfaction with life and achievement. It is possible that the socio-economic landscape of South Africa and the likelihood of most parents being without the requisite skills and resources will cause parental involvement to have a negative effect on the relationship between wellbeing and academic achievement. Using the same TIMSS dataset, Reddy et al. (2022) posit that only 38% of learners reported that one parent has post-secondary school education. It was also reported that 63% of learners had parents who did not understand the language of the home work, while 65% struggled with the content, most likely explaining the emerging negative relationship. Learners whose parents did not struggle with providing homework support were more likely to have higher achievement scores (Reddy et al., 2022). Following the argument by Tam and Chan (2009), parents need to have the requisite skills in order for their involvement in learners’ schoolwork to have a positive impact. Moreover, Grade 9’s are more likely to require specialised support with their homework for any assistance to have the intended outcome of academic success.

#### Satisfaction with life and mathematics achievement mediated by instructional clarity

Given the importance of teacher-learner relationships for academic achievement, we also tested the role of instructional clarity in the association between mathematics achievement and satisfaction with life. We found that instructional clarity partially mediated the hypothesised relationship. This means that the relationship between life satisfaction and mathematics achievement can be partially attributed to the extent to which learners perceive their teacher’s instruction as clear and meaningful. This finding points to how individual-level characteristics (satisfaction with life), together with the situational factor of instructional clarity, predict mathematics achievement. For Grade 9 learners (in the context of the study), evaluating their lives positively and experiencing clarity of teaching in mathematics have the potential of improving academic achievement.

The importance of instructional clarity is emphasised in the fostering of learning practices that increase engagement among learners (Thapa et al., 2013; Lee et al., 2017). Further explanation of the emerging relationship is that clear instruction enables learners to plan more effectively, set goals and acquire a stronger sense of how to judge their own progress—thus fostering achievement. Clarity also allows learners to process course information in elaborate ways that might improve academic outcomes (Bolkan et al., 2016). The mediating role of instructional clarity is further support for positive education as an approach toward enhancing academic achievement while fostering the wellbeing of the learners. Instructional clarity not only addresses the issue of cognitive load arising from the appropriate design of learning materials, it also has the potential of ensuring engagement among learners, which is one of the core aims of positive education (Seligman and Adler, 2018).

### Limitations of the study

Our study has a number of positive implications but is not without limitations. One common limitation of cross-sectional data is the inability to draw causal conclusions. As much as we can argue for the relationship between satisfaction with life and mathematics achievement, there has been evidence for the reverse relationship. Hence, a longitudinal study is still needed to determine the causal directionality of the hypothesised relationship.

In addition, the current study only explored the moderating role of gender and did not control for other confounding variables including SES, race, or province of origin and a host of school-level variables, all of which might influence results. Although beyond the scope of the study, we wonder whether qualitative assessment of satisfaction with life would not provide better information on what aspects of subjective wellbeing require policy-related interventions.

### Practical implications

This study has some practical and research implications for education and wellbeing studies, both locally and internationally. Firstly, our work suggests that fostering subjective wellbeing at schools is worthwhile in developing economies like South Africa, because of its relationship with mathematics achievement. Secondly, we have demonstrated that both the school and home contribute to the relationship between subjective wellbeing and mathematics achievement. Specifically, our findings suggest that instructional practices in South African schools need to be improved by education stakeholders by making the instructional process more and more suitable to learning needs if better achievement scores in mathematics are expected. We also show how such improvements in instructional practices could be accompanied by better wellbeing of learners.

It would seem that certain kinds of parental involvement do not relate to improvement in mathematics achievement. This is most likely in low-income contexts including South Africa, where most parents might not have adequate knowledge and expertise to properly support learners in their school work. As a result, additional governmental assistance might be needed in terms of after-school tutoring to improve achievement in mathematics to supplement parental support.

## Conclusion

The aim of the present study was to explore the nature of the relationship between satisfaction with life and mathematics achievement among Grade 9 South African learners. We also tested the roles of gender, instructional clarity and parental involvement in this relationship. The results of the structural equation modelling showed that satisfaction with life is a predictor of mathematics achievement. This relationship is further enhanced by instructional clarity in the teaching of mathematics. However, parental involvement that does not contribute quantitatively to the learner’s schoolwork emerged as a negative mediator of the relationship between satisfaction with life and mathematics achievement. Subjective wellbeing is important for achievement but must be accompanied by appropriate support from the home and school.

## Data availability statement

The datasets presented in this study can be found in online repositories. The names of the repository/repositories and accession number(s) can be found below: https://www.timss-sa.org/dataset/timss-2019-grade-9-learner-and-school-context-data.

## Ethics statement

The studies involving human participants were reviewed and approved by the Human Sciences Research Council Research Ethics Committee. Written informed consent to participate in this study was provided by the participants or their legal guardian/next of kin.

## Author contributions

AWF: conceptualisation, analysis, methodology, and prepared the original draft. VR: principal investigator for TIMSS 2019 in South Africa, project administration, review, and editing. Both authors contributed to the article and approved the submitted version.

## Footnotes

1 Note that the results for only one plausible value of mathematics achievement are presented in all the tables, instead of the fit indices for each of the five plausible values of mathematics achievement, given that all the fit statistics and coefficients reflect similar findings.
